# Mutations associated with Dent's disease affect gating and voltage dependence of the human anion/proton exchanger ClC-5

**DOI:** 10.3389/fphys.2015.00159

**Published:** 2015-05-19

**Authors:** Alexi K. Alekov

**Affiliations:** Institut für Neurophysiologie, Medizinische Hochschule HannoverHannover, Germany

**Keywords:** Dent's disease, ClC transport, ClC-5, non-linear capacitance, gating currents, vesicular acidification, endocytosis, voltage dependence

## Abstract

Dent's disease is associated with impaired renal endocytosis and endosomal acidification. It is linked to mutations in the membrane chloride/proton exchanger ClC-5; however, a direct link between localization in the protein and functional phenotype of the mutants has not been established until now. Here, two Dent's disease mutations, G212A and E267A, were investigated using heterologous expression in HEK293T cells, patch-clamp measurements and confocal imaging. WT and mutant ClC-5 exhibited mixed cell membrane and vesicular distribution. Reduced ion currents were measured for both mutants and both exhibited reduced capability to support endosomal acidification. Functionally, mutation G212A was capable of mediating anion/proton antiport but dramatically shifted the activation of ClC-5 toward more depolarized potentials. The shift can be explained by impeded movements of the neighboring gating glutamate Glu_ext_, a residue that confers major part of the voltage dependence of ClC-5 and serves as a gate at the extracellular entrance of the anion transport pathway. Cell surface abundance of E267A was reduced by ~50% but also dramatically increased gating currents were detected for this mutant and accordingly reduced probability to undergoing cycles associated with electrogenic ion transport. Structurally, the gating alternations correlate to the proximity of E267A to the proton glutamate Glu_in_ that serves as intracellular gate in the proton transport pathway and regulates the open probability of ClC-5. Remarkably, two other mammalian isoforms, ClC-3 and ClC-4, also differ from ClC-5 in gating characteristics affected by the here investigated disease-causing mutations. This evolutionary specialization, together with the functional defects arising from mutations G212A and E267A, demonstrate that the complex gating behavior exhibited by most of the mammalian CLC transporters is an important determinant of their cellular function.

## Introduction

Dent's disease (Dent and Friedman, 1964) is a X-linked hereditary disease coupled to impaired function of the kidney. The pathophysiology of the disease has been connected to mutations in two different genes – *CLCN5* and *OCRL1* (Lloyd et al., 1996; Hoopes et al., 2005). *OCRL1* encodes a Golgi-localized PI(4,5)P2 5-phosphatase that interacts with clathrin and regulates protein trafficking between endosomes and the Golgi network (Suchy et al., 1995; Zhang et al., 1995; Choudhury et al., 2005). The second gene, *CLCN5*, encodes for the membrane transporter ClC-5 that resides in endocytotic vesicles (Günther et al., 1998; Sakamoto et al., 1999) and mediates secondary active chloride/proton exchange (Picollo and Pusch, 2005; Scheel et al., 2005). Knockout of *Clcn5* in mice is associated with impaired renal endocytosis and significantly slowed rates of endosomal acidification (Piwon et al., 2000; Günther et al., 2003). Similar effects have been observed in conditionally immortalized proximal–tubular epithelial cell lines derived from Dent's disease patients carrying ClC-5 mutations (Gorvin et al., 2013). Recent investigations suggest that ClC-5 might be also involved in the regulation of intraendosomal chloride concentration (Novarino et al., 2010). Impaired endocytosis and endosomal ion homeostasis seem therefore to represent the major mechanisms leading to Dent's disease.

**Figure 1:**
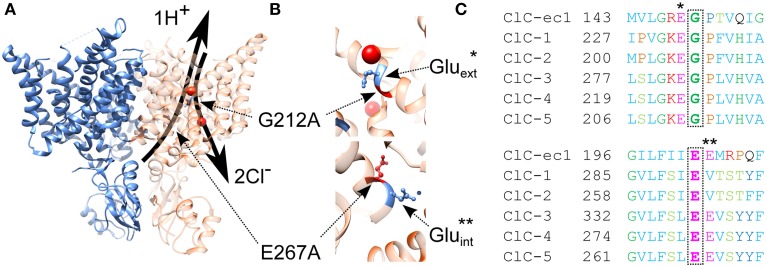
**Structural localization and evolutionary conservation of two Dent's disease associated mutations. (A)** Ribbon representation of the dimeric biological assembly typical for proteins of the CLC family and based on the crystal structure of the eukaryotic CmCLC (PDB id: 3ORG). Two identical monomers capable of mediating coupled chloride/proton exchange are assembled to form a functional CLC dimer. Superimposed as arrows on the right monomer are the anion and proton transport pathways, separated at the bottom (intracellular) side and converging at the central anion biding site to follow the same exit/entry pathway toward the top of the protein. The two here investigated Dent's disease mutations are localized at key positions along these pathways—mutation G212A is located at the top of the protein at the separate exit for anions and protons; mutation E267A is localized at the intracellular entrance of the proton transport pathway. **(B)** Enlarged view of the protein regions surrounding mutations G212A and E267A with additionally annotated the gating [Glu_ext_ – indicated with a star here and in the sequence alignment in **(C)**] and proton [Glu_in_ – indicated with two stars here and in the sequence alignment in **(C)**] glutamates that both play decisive roles for the CLC transport cycle. **(C)** Sequence conservation of the regions surrounding both investigated Dent's disease mutations. The glycine G212 is an amino acid that is conserved throughout the mammalian CLC isoforms and precedes the gating glutamate Glu_ext_. Glutamate E267 is also well-conserved and precedes the proton glutamate E268 in the sequence of ClC-5.

The clear association between genetic alternations in *CLCN5* and Dent's disease has motivated numerous investigations of the molecular mechanisms underlying the renal pathophysiology observed in the affected patients. The functional consequences of the majority of the currently mapped mutations have been already described. Surprisingly, the data suggest the existence of very significant phenotypic heterogeneity with one aspect of this heterogeneity appearing especially interesting. In particular, most of the mutants (class 1 mutants) have been found to induce a trafficking defect to the plasma membrane which reduces the electrogenic transport mediated by ClC-5 as detected by electrophysiology (Ludwig et al., 2005; Smith et al., 2008; Grand et al., 2009, 2011). However, a distinct subclass of mutants have been also described for which ion transport is strongly reduced or even completely abolished despite the significant number of ClC-5 proteins present in the plasma membrane. The molecular mechanisms underlying this behavior have not been revealed yet; however, the complex nature of the CLC transporter operation allows several possible explanations (Smith et al., 2008; Grand et al., 2009, 2011; Lourdel et al., 2012). For example, the corresponding mutations could block the ion permeation pathway, alter the transporter selectivity and substrate coupling or reduce unitary transport rates of ClC-5. In this regard, prominent voltage-dependent gating has been described as a hallmark feature exhibited by most of the mammalian CLC isoforms (Alekov and Fahlke, 2009; Smith and Lippiat, 2010; Orhan et al., 2011; Grieschat and Alekov, 2012; Guzman et al., 2013; Stefano et al., 2013). It is well-established that altered voltage-dependent gating plays a major role for the pathophysiology of various hereditary diseases associated with members of the “channel branch” of the CLC family. It appears therefore very likely that analogous effects might be involved in the development of Dent's disease and that alternations of the voltage dependence of ClC-5 might be responsible and explain the reduced current amplitudes observed in this particular subclass of mutants for which no change in surface expression is detected. Here, this hypothesis is tested by investigating the functional consequences of two point mutations that have been previously associated with Dent's disease by genetic analysis. The first one, G212A, has been shown to reduce ClC-5 current amplitudes without altering its surface abundance (Grand et al., 2009). The choice of this particular mutation was motivated by its close proximity to the so-called gating glutamate E211 that is crucial for voltage-dependent gating of both CLC channels and transporters (Glu_ext_, Figure 1) (Dutzler et al., 2003). It appears therefore possible that G212A might affect ClC-5 voltage sensing and lead in this way to reduced CLC transport. For the second Dent's mutation, E267A (Hoopes et al., 2004), no functional investigations have been published until now. Similarly to G212A, mutation E267A is close to a residue that plays an important role in the CLC transport cycle. In particular, it is next to the so called proton glutamate E268 (Glu_in_) that serves as a gate for protons from the intracellular side of the CLC protein (Accardi et al., 2005) and defines the apparent transport probability of ClC-5 by regulating proton injection into the ClC gating machinery (Grieschat and Alekov, 2012). Strikingly, some of the CLCs do not have a glutamate at the corresponding position, i.e., they lack the proton glutamate Glu_in_ but are still capable of transporting protons in exchange for chloride. It was therefore hypothesized that the neighboring glutamate which corresponds to the Dent's E267 in ClC-5 (Figure 1) might take over the role of Glu_in_ and enable coupled anion/proton in these isoforms (Feng et al., 2010; Phillips et al., 2012). The role of glutamate E267 in the mammalian transporter isoforms has not been investigated until now; however, it appears feasible that this residue and accordingly the molecular mechanisms underlying the pathophysiology of the Dent's disease mutation E267A might be also coupled to proton transport in ClC-5.

## Materials and methods

### Constructs and functional expression

The construction of the pRcCMV vector containing the DNA encoding for human ClC-5 with the fluorescent protein mCherry fused to its C-terminus was described previously in detail (Grieschat and Alekov, 2012). The YFP fusion used in some of the experiments was also created previously in an identical manner. The Dent's disease mutations were introduced into the mCherry-containing construct using QuikChange site-directed mutagenesis (Agilent) and verified by sequencing. An extracellular haemagglutinin (HA)-tag was inserted into the mCherry construct in the ClC-5 sequence between amino acids 392 and 393. This position is predicted to be situated at one of the long extracellular loops in mammalian CLCs and was used previously to assess the surface expression of ClC-2 (Garcia-Olivares et al., 2008). The synapto-pHluorin2 construct was a gift from Dr. Guzman and was created by replacing the pHluorin in the original synapto-pHluorin, kindly provided by Dr. Miesenböck (Miesenböck et al., 1998), for the brighter pHluorin2 (Mahon, 2011) that was a gift from Dr. Mahon, and subcloning the resulting fusion construct into the p156rrL vector using standard PCR procedures. mRFP-Rab5 was a gift from Ari Helenius (Addgene plasmid # 14437) (Vonderheit and Helenius, 2005). HEK 293T cells used for the experiments were cultured in DMEM (Gibco) supplemented with 10% FBS (Biochrom AG), 2 mM L-glutamine and 50 units/ml penicillin/streptomycin (Invitrogen). When required, cells were transfected using the calcium phosphate precipitation method (Graham and van der Eb, 1973).

### Electrophysiology

Electrophysiology was performed as previously described in detail (Grieschat and Alekov, 2012). In brief, EPC-10 amplifier, controlled by the PATCHMASTER software package (both from HEKA Electronics), was used to perform whole-cell patch-clamp (Hamill et al., 1981) with currents being recorded after filtering at 3 kHz and digitalization at 100 kHz sampling rate. To reduce the associated voltage errors, capacitance cancelation and series resistance compensation were applied and recordings for which the uncompensated error exceeded 5 mV were discarded. Patch pipettes with resistances between 1 and 1.8 MΩ were filled with a patch pipette solution containing (in mM) 110 NaCl, 5 MgCl_2_, 5 EGTA and 10 HEPES, pH 7.4. The standard extracellular solution contained (in mM) 145 NaCl, 4 KCl, 2 CaCl_2_, 1 MgCl_2_, and 15 HEPES, pH 7.4. In some cases, P/4 leak subtraction (Armstrong and Bezanilla, 1974) was applied from a holding potential of −60 mV. Non-linear capacitances were measured using the Lock-in extension of PATCHMASTER (HEKA Electronics). In particular, the sine-plus-DC technique (Gillis, 2000) was used with 10-mV sine waves with 400-Hz frequency being superimposed on DC pulses of variable voltage. When appropriate (for WT and mutation E267A ClC-5), non-linear capacitances were fitted with the first derivative of a standard Boltzmann function (Santos-Sacchi, 1991):

(1) $$C(V)=\frac{\beta Q_{\max}\;e^{-\beta (V_{0}-V_{0.5})}}{{\left(1+e^{-\beta (V_{0}-v_{0.5})}\right)}^{2}}with\;\beta =z\left(\frac{e_{0}\delta }{k_{B}}T\right)$$

where *Q_max_* denotes the maximum charge moved at the voltage of half-maximal activation (*V*_0.5_), *z* represents the number of elementary charges *e*_0_ displaced over a membrane fraction (δ), *k_B_* is the Boltzmann constant and *T* is the absolute temperature.

### Confocal imaging and vesicular pH measurements

Images were acquired 24-48 h after transfection on a Carl-Zeiss LSM 780 inverted microscope using a 40x water immersion objective or on an ANDOR spinning disk imaging system equipped with Yokogawa CSU-X1 unit using a 60x water immersion objective. The pHluorin2 and mCherry fluorophores were excited at 405/488 and 561 nm and emission was detected at 500-550 and 560-650 nm, respectively. The fluorescence of mRFP and YFP was excited by the 561-nm and the 514-nm laser lines, respectively, and detected by an ANDOR IXON3 camera. For the colocalization analysis of Rab5-RFP and ClC-5-YFP or synapto-pHluorin2, a CAIRN OptoSplit mage splitter was used to separate the fluorescence of the individual fluorophores. Cells were maintained during live cell imaging in PBS containing Ca^2+^ and Mg^2+^ (GIBCO) at room temperature (22–24°C). For the calibration experiments, PBS was exchanged for potassium-based solutions with different pHs and supplemented with 10 μM nigericin. The analysis of the calibration data and the assembly of the confocal images for publication were performed using Carl Zeiss Zen lite 2011 (Blue edition) software. Particle detection was performed using the MatLab (MathWorks) adaptation by Blair and Dufresne of the original Crocker and Grier algorithm (Crocker and Grier, 1996). The code was incorporated into house-written MatLab software that carried out the automatic background subtraction, segmentation using the before-mentioned algorithm, and the subsequent ratiometric analyses of the identified vesicular regions. The colocalization between Rab5-RFP and ClC-5-YFP or synapto-pHluorin2 was determined using the standard colocalization analysis plugin of IMAGEJ (Rasband, n.d.).

### Relative surface expression of ClC-5

The relative surface expression of WT and mutant ClC-5 was determined in cells transfected with HA-ClC-5 (see below) using a slight modification of previously published methods (Smith et al., 2008). In brief, the ClC-5 proteins localized at the surface membrane were labeled in suspension at 4°C for 1 h with anti-HA antibodies targeting an extracellular HA tag engineered in one of the predicted extracellular loops of mammalian CLC proteins (Garcia-Olivares et al., 2008), washed three times with cold PBS and subsequently labeled with anti-mouse horseradish perioxidase (HRP)-conjugated antibodies (all antibodies were obtained from Thermo Scientific). The amount of cell-bound HRP was determined after additional extensive washing by chemi-luminescence using a VICTOR3 plate reader (PerkinElmer). To obtain the relative surface expression of the investigated constructs, the HRP-coupled luminescence was divided by the fluorescence of the C-terminal mCherry tag that was excited at 488 nm and measured at 535 nm wavelength in the same well and reported the total ClC-5 expression.

### Fluorescence measurements of intracellular pH

Measurements of intracellular alkalinization as marker for the CLC proton transport were described in detail elsewhere (Alekov and Fahlke, 2009). In brief, cells were loaded with 37.5 μM 2′,7′-bis(2-carboxyethyl)-5(and 6)-carboxyfluorescein (BCECF, Wako Chemicals) through the patch pipette. The HEPES content of the intracellular patch-clamp solution was reduced in these experiments to 0.25 mM in order to reduce its buffering capacity. BCECF fluorescence was observed with an UPlanSApo 60x/NA1.35 oil immersion objective on an Olympus IX-71 microscope. Sequential excitation at 490 and 440 nm was applied using a Polychrome V monochromator and the fluorescence was detected at 530 nm with a photodiode (both from Till Photonics). The resultant fluorescence ratio F490/F440 was converted to absolute pH by using a calibration curve, previously obtained *ex situ* (see description in Grieschat and Alekov, 2012). The rate of cytosolic alkalinization (ΔpH/Δ*t*) was obtained from linear fits to the data and used as a value proportional to the proton flux of ClC-5.

### Data analysis and presentation

Data were analyzed by a combination of Excel (Microsoft), Origin (MicroCal), and MatLab (MathWorks) and assembled as publication figures in Origin. Differences were tested for significance using two-sample *t*-test, all data are presented as mean ± SEM.

## Results

### Mutations G212A and E267A provide weaker support for endosomal acidification in HEK293T cells

**Figure 2:**
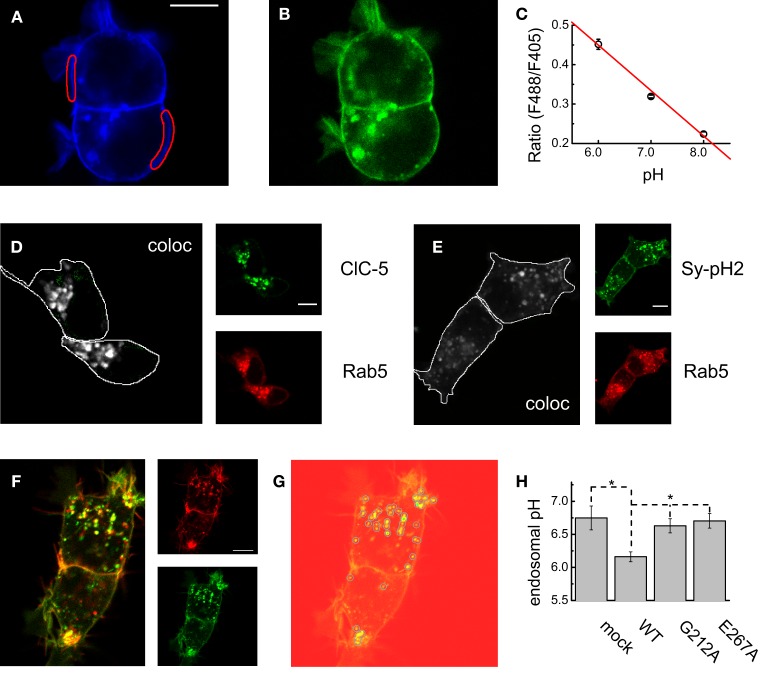
**Luminal pH in WT and disease-associated ClC-5-containing endosomes. (A,B)** Representative confocal images of cells expressing synpto-pHluorin2 excited at 405 nm **(A)** and 488 nm **(B)** and both detected at a wavelength of 530 nm. For the analysis, membrane regions as depicted in **(A)** were selected and the fluorescence intensity in both channels was measured. **(C)** Calibration curve constructed from measurements as depicted in **(A,B)**. The ratios were obtained by taking confocal images from cells (*n* = 6 cells for each experimental point) bathed in solutions with pH-s as indicated and containing nigericin to equilibrate the extracellular and intracellular acidity. The calibration curve was constructed by fitting a straight line to the data. **(D)** Representative cellular distribution and map of the colocalizing pixels for coexpressed Rab5-RFP and ClC-5-YFP expressed in HEK293T cells. **(E)** Representative cellular distribution and map of the colocalizing pixels for coexpressed Rab5-RFP and synapto-pHluorin2 expressed in HEK293T cells. **(F)** Representative confocal images as used for determining intravesicular pH. The large image represents the overlay of the fluorescence using 560 nm laser line to excite the mCherry attached to ClC-5 (red) and the 488-nm laser line to excite the synapto-pHluorin2 (green) molecules. Both channels are represented as small sub-images at the right of the overlay; for simplicity, the 405-nm channel was not depicted. **(G)** Illustration of the particle identification procedure used to select individual vesicular regions in the red channel (ClC-5-containing endosomes) and used to measure the fluorescence intensities in both pHluorin2 channels. The identified particles are overlaid as circles on the red channel of the cells depicted in **(F)**. For measuring of endosomal pH in cells not transfected with ClC-5, particle detection was performed analogously using the florescence images taken in the blue channel (405-nm excitation). **(H)** Average vesicular pH determined as depicted in **(F,G)** for cells transfected with synapto-pHluorin2 only (mock, *n* = 7) or cotransfected with synapto-pHluorin2 and either WT, G212A or E267A ClC-5 (*n* = 18, 18, 9, respectively). First, the ratio of the intensities of the blue and green channels (excitation at 405 and 488 nm, both detected at 530-nm wavelength) was calculated and this ratio was subsequently converted to absolute pH using the calibration curve depicted in **(C)**. Significant differences at the level of 0.05 are indicated as stars.

The role of ClC-5 for endosomal acidification is well-established and is of crucial importance for the proper function of the kidney (Jentsch, 2008). The first step of the investigation of both disease causing mutations was therefore to investigate their capacity to support endosomal acidification. To this end, the ratiometric pH-sensitive GFP variant pHluorin2 (Mahon, 2011) was fused to the C-terminus of the vesicular protein VAMP-2/synaptobrevin in analogy to the original synapto-pHluorin construct developed for vesicular pH measurements (Miesenböck et al., 1998). In this way, a pH-sensitive fluorophore with superior brightness was targeted to the vesicular lumen. Synapto-pHluorin2 was excited at two different wavelengths (405 and 488 nm) and the fluorescence intensity at the 530-nm pHluorin2 emission maximum was measured on a confocal microscope. 2D-imaging was chosen for these investigation in order to reduce the time lag between capturing the fluorescence excited at different wavelengths and accordingly the spatial shift between the different channels due to the motility of the endocytotic vesicles. A calibration curve that compares the ratio of the fluorescence excited at the two wavelengths (the ratio F488_ex_/F405_ex_) and the absolute pH sensed by the fluorescent protein was constructed by performing experiments in solution with different pH-s and clamping the intracellular acidity with the H^+^/K^+^ exchanger nigericin (Figures 2A–C). For these experiments, live cells that exhibited strong plasma membrane localization of synapto-pHluorin2 were chosen and exclusively defined membrane regions were selected and analyzed (Figures 2A,B). At the next step, the colocalization of ClC-5 and synapto-pHluorin2 was demonstrated by coexpression with the endosomal small GTPase Rab5 (Figures 2D,E). Finally, the pH of vesicles containing either ClC-5 WT or the mutants G212A and E267A was measured by automatic vesicle identification (Figures 2F–H) and ratiometric fluorescence measurements. Live cells expressing both ClC-5 and synapto-pHluorin2 were imaged sequentially at three different wavelengths – 561 nm to excite the red mCherry attached to ClC-5 and 488 and 405 nm to excite the different spectral lines of pHluorin2 (Figure 2F). ClC-5-containing vesicles were identified in the red channel by using automated spot detection routines (Figure 2G) and the ratio F488_ex_/F405_ex_ measured in the so-defined circular regions was used to quantify endosomal pH. The analysis revealed that expression of WT ClC-5 leads to significantly stronger endosomal acidification in HEK293T cells. In contrast, there was no difference between cells expressing synapto-pHluorin2 alone or together with either of both ClC-5 variants associated with Dent's disease (Figure 2H). The encountered significant differences suggest that the capacity of ClC-5 to support endosomal acidification is compromised by the here investigated disease-causing mutations and provide the bases for understanding of the associated renal pathophysiology.

### Mutations G212A and E267A exhibit similar mixed endosomal/plasma membrane localization and different transport properties in comparison to WT ClC-5

**Figure 3:**
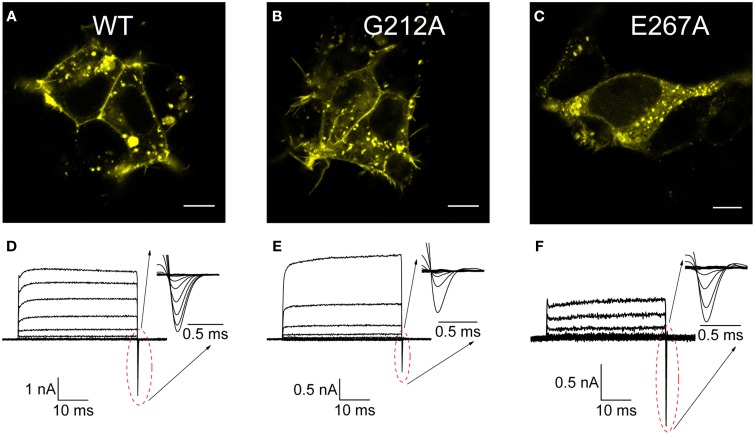
**Cellular localization and transport of WT and disease-associated ClC-5 mutants. (A–C)** Confocal florescent images of cells transfected with either WT or the mutants G212A and E267A ClC-5 with fused mCherry at the C-terminus. Visible as bright fluorescent spots is the strong vesicular localization but a significant percentage of the ClC-5 proteins are also localized to the plasma membrane. **(D–F)** Current families recorded from cells expressing the investigated constructs by whole-cell patch-clamp upon voltage steps between −115 and +175 mV. The insets depict enlarged the off-gating currents for the corresponding mutant that have been used subsequently for estimating the voltage-dependence of ClC-5 activation.

The next step of the investigation of mutations G212A and E267A ClC-5 was to describe their functional properties and to establish a link between these and the encountered reduced vesicular acidification. To this end, 3D confocal imaging was performed first to investigate in further detail the cellular distribution of both Dent's mutations. The images showed that all investigated constructs exhibit similar mixed localization pattern. The majority of the ClC-5 proteins resided in vesicular structures but also a significant plasma membrane localization was evident (Figures 3A–C). This allowed performing in a next step an electrophysiological characterization of the CLC anion/proton exchange by whole-cell path-clamp. The well-defined current trace families obtained upon voltage steps between −115 and +175 mV (Figures 3D–F) demonstrate that both investigated mutants did not lose their ion transport capability. However, despite the overall similarity to the WT ClC-5, these measurements revealed also that the mutants exhibit significantly smaller transport current amplitudes. Moreover, while both G212A and E267A exhibited prominent gating currents (gating charge movements, see insets in Figures 3D–F), their amplitudes in relation to the corresponding transport currents strongly differed from the ones of WT ClC-5. The gating currents of G212A were smaller than the one of WT ClC-5 but mutation E267A exhibited the opposite behavior. A summary of these observation is provided in Figure 5. Since gating currents in ClC-5 reflect the existence of incomplete silent transport cycles, i.e., conformational changes that are not associated with electrogenic chloride/proton exchange (Grieschat and Alekov, 2012), the encountered differences suggest that the probability for completing the transport cycle with ions being transported through the membrane is altered by both here investigated Dent's disease mutations.

**Figure 4:**
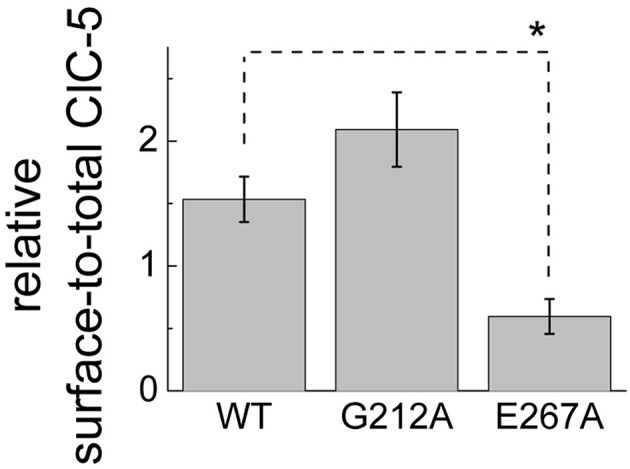
**Relative surface expression of WT and disease-associated ClC-5 mutants**. Ratio of the surface ClC-5 assessed by HRP chemi-luminescence from an antibody targeting a HA tag engineered in one of the extracellular ClC-5 loops (proportional to the surface expressed ClC-5) and the fluorescence of the mCherry bound to the C-termini of the investigated constructs (proportional to the total ClC-5 expression). The significant difference between WT and E267 ClC-5 at the level of 0.05 is indicated by a star.

To quantify the relative surface expression of the mutants and compare it with the encountered reduced ion transport, they were introduced into a previously created construct containing ClC-5 that was C-terminally tagged with mCherry and an HA tag engineered in one of the predicted extracellular loops of the protein. An insertion at the analogous position in ClC-2 was previously successfully used to determine the surface expression of this isoform (Garcia-Olivares et al., 2008). Similarly to ClC-2, the introduced additional amino acids did not alter the properties of ClC-5 (data not shown). The relative number of ClC-5 localized to the plasma membrane was determined by the binding of anti-HA antibodies (surface marker), normalized to the mCherry intensity that was used as a marker for the total ClC-5 expression. The experiments confirmed the previously reported unchanged surface expression of mutation G212A (Figure 4) (Grand et al., 2009) but also showed a significantly lower (~50% compared to WT) plasma membrane localization for the E267A mutation.

### Mutations G212A and E267A alter gating and voltage dependence of ClC-5

**Figure 5:**
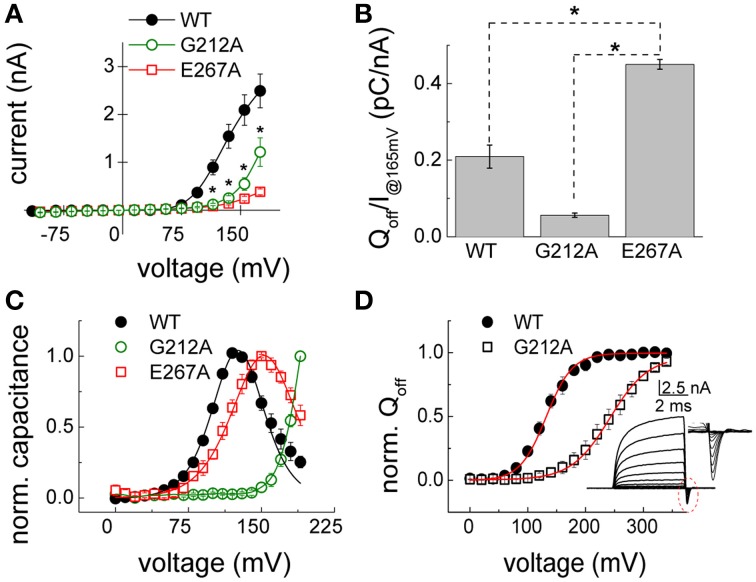
**Gating properties of the disease associated mutations G212A and E267A ClC-5. (A)** Averaged current–voltage relations constructed by measuring the steady-state current amplitudes at the end of voltage steps in the indicated range as depicted in Figures 3D–F for cells expressing either WT (*n* = 5), G212A (*n* = 6) or E267A (*n* = 11) ClC-5. Significant differences (for both mutants vs. WT ClC-5) at the level of 0.05 are indicated as stars. **(B)** Ratio between the off-gating charge (Q_off_) and ionic currents (I) at +165 mV [*n* = 5.11, same cells as in **(A)**]. Significant differences at the level of 0.05 are indicated as stars. **(C)** Voltage dependence of the non-linear capacitances of WT and G212A and E267A ClC-5 determined by using lock-in based impedance measurements. The curves for E267A and WT ClC-5 exhibit the typical bell-shaped form and were fitted with the first derivative of a standard Boltzmann function with the peak corresponding to the half-maximal activation (E267A ClC-5 - *V*_0.5_ = 153 ± 2 mV, *n* = 5; WT ClC-5 - *V*_0.5_ = 128 ± 2 mV, *n* = 5). The curve for mutation G212A did not exhibit a peak within the investigated range and was not fitted. **(D)** Voltage dependence of the activation of mutation G212A WT as obtained from gating current measurements. The inset depicts a representative recording from WT ClC-5 measured with short pulses. The encircled region is additionally depicted enlarged to show the off-gating currents that were integrated to obtain the gating charge Q_off_. The red lines represent Boltzmann fits to the data to obtain the half maximal voltage of activation (G212A and WT ClC-5, respectively, *V*_0.5_ = 240 ± 10 mV, *n* = 4 and *V*_0.5_ = 132 ± 6 mV, *n* = 4).

To further evaluate differences in the behavior of the here investigated Dent's disease mutants, their characteristic current–voltage relations were compared by plotting the transport current amplitudes against the applied voltage. The so-obtained quantitative data confirmed the preliminary observations that current densities of both G212A and E267A ClC-5 are significantly smaller than the one of WT ClC-5 in a broad range of positive voltages (Figure 5A). Moreover, the current reduction for E267A was much higher (~10-fold at +165 mV) than the expected ~two-fold reduction extrapolated from the reduced surface expression of this particular mutant (see Figure 4). In a next step, the macroscopic differences of the gating currents of the mutants were therefore also quantified by comparing their gating charge and transport currents at +165 mV (Figure 5B). In particular, the gating charge was calculated by integrating the surface under the gating currents (see insets in Figures 3D–F) and the resulting value was divided by the ionic currents measured at the same voltage to provide apparent gating charge amplitudes for the investigated constructs. The analysis showed that the activation of mutation E267A mobilizes more gating charge than WT ClC-5 and that mutation G212A exhibits the smallest gating charges from all investigated constructs (Figure 5B). Based on the inverse dependency between apparent gating charge and transport probability of ClC-5, these differences suggest that the probability for undergoing a silent non-transporting cycle (Grieschat and Alekov, 2012) is decreased by mutation G212A but increased by mutation E267A. Put in the context of the Dents' disease pathophysiology, the increased gating charge and accordingly the strongly reduced probability for electrogenic cycling seems sufficient to explain the smaller current amplitudes of mutation E267A ClC-5. This is however in stark contrast to the reduced apparent gating charge and increased open probability encountered for G212A ClC-5. Viewed mechanistically, such increase does not correlate to the actually measured higher endosomal pH in cells expressing this mutant because it should lead to larger current amplitudes and accordingly should favor stronger endosomal acidification. A reduced surface expression or altered cellular localization of this mutant were also dismissed as major defects based on the obtained confocal images, the test of its relative surface expression (see Figures 2–4) and previously published data (Grand et al., 2009). It appeared therefore possible that further gating alternations exist that reduce ion transport of G212A ClC-5. Moreover, indications for such changes are readily notable when observing the macroscopic current families recorded from cells expressing G212A ClC-5 (Figure 3). In particular, the spacing (or change of the amplitude) between the consequent current traces in the whole cell currents of G212A is much larger when compared to the other two investigated constructs. To provide a quantitative measure for this observation, non-linear capacitances were measured (Figure 5C) which are directly related to the gating currents but also report the voltage dependence of the activation of ClC-5 transport (Grieschat and Alekov, 2014). The non-linear capacitances of WT ClC-5 present themselves as a bell-shaped curve with a maximum at around +130 mV and can be described mathematically by a function obtained as the first derivative of a standard one-step Boltzmann activation. The peak of the curve corresponds to the voltage of half-maximal activation of the process, i.e., the voltage at which an apparent probability of 0.5 for ClC-5 to activate and undergo a cycle with electrogenic ion transport is reached. Performing analogous measurements on both Dent's disease mutants revealed different behavior. The non-linear capacitances of E267A behaved in the expected way and provided a bell-shaped curve. Their voltage dependence was slightly shifted to stronger depolarized potentials, a change that will also lead to a reduction of the expected ClC-5 ion transport for this particular mutant. On the contrary, the non-linear capacitances of G212A did not produce a peaked curve within the investigated voltage rage up to +190 mV but increased steeply with voltage starting at around +150 mV. This behavior suggests that the activation of G212A is dramatically shifted to the right, but unfortunately also precludes obtaining precise quantitative information on the magnitude of this shift. The here used lock-in capacitance measurements require applying AC sinus voltage with a frequency of 400 Hz that is superimposed on DC steps with voltages covering the range of interest. Moreover, data of many sinus repetitions are averaged in order to increase the precision of the measurements. This requires using long voltage steps and precludes extending the investigated voltage range because at high voltages the whole-cell configuration becomes rapidly destabilized. To overcome this difficulty, the voltage dependence of G212A was assessed by analyzing the gating charge mobilized upon application of depolarizing voltage steps. For the purpose of this analysis, the measurement protocol used to assess whole-cell currents and gating charges of ClC-5 was optimized. In particular, very short pulses (5-ms long) were used which preserved stable whole-cell configuration for voltages as high as +350 mV. Integrating the off-gating currents obtained using this protocol provided an estimate of the voltage dependence of G212A because the gating charge saturated also for this mutant at high voltages and accordingly could be described as a standard two-state Boltzmann activation (Figure 5D). The analysis showed that the activation of G212A is shifted by ~100 mV to the right when compared to WT ClC-5 and measured under analogous conditions. The encountered dramatic shift suffices to explain the reduced transport activity and accordingly the reduced potency of the mutant to support endosomal acidification (see Figure 2).

### Mutation G212A does not uncouple proton from anion transport

**Figure 6:**
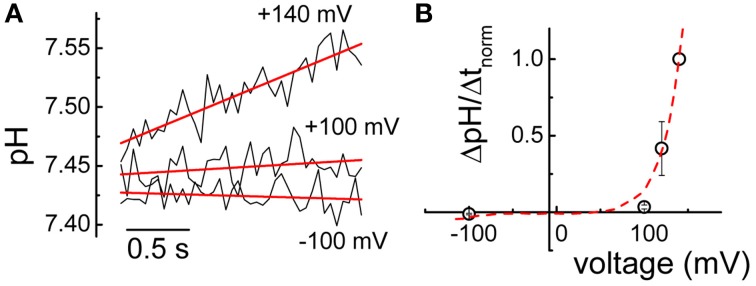
**Proton transport mediated by mutation G212A. (A)** Representative alkalinization recorded at different voltages in a cell transfected with mutation G212A ClC-5 using ratiometric BCECF fluorometry. Lines represent linear fits to the data and were used to provide the rates of alkalinization ΔpH/Δ*t*. **(B)** Averaged rates of alkalinization normalized to the value measured at +140 mV (*n* = 5). The red line represents the current–voltage relation of G212A ClC-5 depicted in Figure 5A and analogously normalized to the current measured at +140 mV.

Both here investigated Dent's mutations reside in the immediate proximity of either the gating Glu_ext_ or the proton Glu_in_ glutamate (see Figure 1). Since neutralization of the negative charge of these residues have been shown to completely uncouple proton from anion transport in ClC-5 (Zdebik et al., 2008), the possibility that the here investigated Dent's disease mutation are associated with similar defects has been also tested. Similarly to previously described experiments (Alekov and Fahlke, 2009), cells expressing both mutants were loaded with the pH-sensitive dye BCECF trough a patch pipette under whole-cell patch clamp and the intracellular alkalinization upon depolarizing voltage steps was measured by ratiometric fluorometry. For mutation E267A, no specific (that differed from measurements in untransfected cells) proton flux could be detected even at the highest investigated voltage of +140 mV (data not shown). This finding should however be taken with care because the dramatically reduced transport probability of E267A might bring its proton flux below the experimental resolution. In contrast, significant voltage-dependent alkalinization could be recorded in cells expressing G212A ClC-5 (Figure 6A) which shows that similarly to WT ClC-5 (Grieschat and Alekov, 2012), chloride transport is coupled to proton antiport in this mutant. Moreover, superimposing the relative rates of intracellular pH changes on the normalized current–voltage relation revealed that they exhibit identical voltage dependence (Figure 6B). It seems therefore that the shifted ClC-5 activation represents the major defect underlying the pathophysiology of mutation G212A.

## Discussion

### Type 3 dent's disease mutations impair vesicular acidification

Several classifications of the various functional phenotypes exhibited by the already described Dent's disease mutations have been created to group the individual mutations in accordance to the identified functional defects (Smith et al., 2008; Lourdel et al., 2012). Despite the partially overlapping characteristics of the different mutations, their classification can be summarized as follows: Class 1 mutations impair the processing and folding of ClC-5 and are characterized by a pronounced retention in the endoplasmic reticulum; Class 2 mutations induce a delay or change in other way the protein processing which alters the predominant cellular localization of the ClC-5 proteins although they seem to leave the ER in a fully functional form; Class 3 comprises a subset of mutations that seem to exhibit high level of surface abundance when expressed in mammalian cells or *Xenopus laevis* oocytes but to significantly reduced electrogenic CLC transport. Remarkably, no clear correlation between structural localization of the identified mutations and their functional phenotype has been established until now. Here, this problem has been addressed by investigating two Class 3 mutations with the aim of providing insight into the mechanism that underlie their reduced transport activity. The first one, G212A, was already functionally tested by another group and classified as Class 3 mutation (Grand et al., 2009). The second one, E267A (Hoopes et al., 2004), has not been functionally expressed until now. While surface expression for this mutant was significantly reduced when compared to WT ClC-5 (Figure 4, ~two-fold), the much stronger ionic transport reduction (~10-fold) suggests that E267A also belongs or shares an overlapping phenotype with the Class 3 group of mutants. Fluorescence based measurements of vesicular acidity showed that both mutations are significantly less efficient at supporting endosomal acidification when compared to ClC-5 WT (Figure 2). These measurements at the cellular level provide the mechanistic bases for the pathophysiology observed in the affected patients and correlate neatly with the currently accepted role of ClC-5 in the regulation of endosomal pH (Jentsch, 2008). It can be therefore concluded that similarly to mutations belonging to the other classes, Class 3 mutations impair vesicular acidification in kidney epithelia.

### Correlation between functional phenotype and structural localization of ClC-5 mutations associated with dent's disease

Electrophysiological and imaging investigations showed that both here investigated mutants display mixed plasma membrane and vesicular cellular localization and are capable of mediating transport currents but that the amplitudes of these currents are dramatically changed (Figures 3–5). These results confirmed previously published findings for mutation G212A (Grand et al., 2009) and showed also that mutant E267A, that has not been functionally investigated previously, causes even larger transport reduction. However, the effects responsible for this reduction are very different. Mutation G212A dramatically shifts the activation of ClC-5 toward more depolarized potentials by more than 100 mV (Figures 5C,D). In essence, this means that much higher voltages are required to activate this mutant. Analyzing the amino acid sequence around this particular mutation shows that it changes one of the two glycine residues that surround the gating glutamate Glu_ext_ (E211 in ClC-5, see Figure 1). This glutamate has been demonstrated to play a crucial role for the CLC transport cycle. Crystallographic studies have shown that depending on its protonation status, it can undergo large conformational changes and occupy one of the two upper-most of the three anion binding sites forming the CLC anion selectivity filter or reside in the extracellular solution (Dutzler et al., 2003; Feng et al., 2010). Such movements require that the participating region possesses a relatively large flexibility. It seems therefore that the role of the two glycines on both side of of Glu_ext_, one of which is altered by the here investigated disease-causing mutation G212A, is to provide this flexibility. Accordingly, the exchange of G212 for alanine reduces this flexibility and impedes the the movement of the Glu_ext_ from the external solution into the selectivity filter, an effect that correlates with the significantly higher energy required to activate ClC-5 (Figure 5). It is interestingly to note that a mutation at the analogous position in *CLCN1*, G233S (Richman et al., 2012), was associated with dominantly inherited myotonia congenita. Mutations of G233 exert very different effect on the gating of ClC-1 and lock the channel in the open conformation at negative potentials (Fahlke et al., 1997; Richman et al., 2012). This is in good correlation with the findings reported here for G212A and reflects the fact that, in contrast to ClC-5, Glu_ext_ in ClC-1 has to move out of the selectivity filter upon activation in order to clear the entrance of the selectivity filter and allow diffusive chloride flux.

Mutation E267A also reduces the ClC-5 transport and shifts its activation to the right. However, the shift is much smaller when compared to G212A which contrasts the encountered much larger current amplitude reduction (see Figures 3, 5). Similar small shifts of the depolarization-induced activation have been previously described for other ClC-5 mutations associated with Dent's disease and have been dismissed as causative for the disease pathophysiology because they similarly did not correlate with the observed current amplitude reduction (Gorvin et al., 2013). The findings reported here provide explanation for these phenomena by showing that an additional, very different mechanism contributes to the current reduction observed for mutation G267A. In particular, the apparent gating charge is increased for this mutant which results in gating currents that in comparison to ClC-5 WT are much larger when normalized to the ion transport at the same voltage (Figure 5B). Previous investigations have shown that charge movements in mammalian CLC transporters originate from silent transport cycles that fail to complete because of the insufficient delivery of intracellular protons (Grieschat and Alekov, 2012, 2014; Guzman et al., 2013). It appears therefore reasonable to conclude that mutation E267A also impairs proton delivery and reduces in this way the probability of ClC-5 to undergo a cycle associated with electrogenic ion transport. This hypothesis is supported by the structural proximity of E267 to the proton glutamate Glu_in_ (E268 in ClC-5, see Figure 1) that has been postulated to form the entrance for intracellular protons. Moreover, it has been previously postulated by others that the glutamate next to Glu_in_, which corresponds to the here investigated position E267, also represents a key player in the process of CLC proton transport. In particular, two other CLC isoforms have been shown to mediate coupled anion/proton transport despite the lack of a negatively charged residue at the position of the proton glutamate Glu_in_ (Feng et al., 2010; Phillips et al., 2012). This led to the hypothesis that the glutamate corresponding to the Dent's disease position E267 can take over the role of Glu_in_. The data reported here substantiate this hypothesis and demonstrate that the glutamate E267, similarly to the proton glutamate Glu_in_, regulates the injection of intracellular protons into the interior of ClC-5. This demonstrates therefore that glutamate E267 represents a key element of the CLC transport cycle also in the mammalian CLC transporters. It is interesting to note that BCECF-based experiments failed to detect proton transport for mutation E267A. It is therefore possible that in addition to the reduced current amplitude, this mutation is associated with partial transport uncoupling and permits limited anion flux without the counterflux of protons. This hypothesis also has its attractiveness because coupled anion/proton exchange rather than chloride conductance has been recently shown to be crucial for renal endocytosis (Novarino et al., 2010). If correct, this would imply the existence of an additional differences in the phenotype exhibited by the here investigated mutants. As a consequence, proximal tubule cells expressing the E267A mutant should exhibit more severe endocytosis defects. It appears therefore interesting to address in the future the unclear transport coupling stoichiometry of mutation E267A and the question whether this is coupled to endosomal defects that resemble the phenotype encountered in mice for which ClC-5 coupling has been abolished by neutralizing the gating glutamate. The uncoupling assumption should, however, be taken with care at this point because it is also possible that due to the dramatically reduced ClC-5 currents, proton transport of mutation E267A is below the level of resolution of the technique used here to assess intracellular alkalinization.

### Physiological implications

Voltage-dependent gating has been very early established as an important determinant of the physiology of the channel members of the CLC family (Jentsch, 2008). Surprisingly, such correlation seems to be lacking for ClC-5. Recently, an accelerated gating kinetics of the lysosomal Cl/H-exchanger ClC-7/Ostm1 has been brought up as the reason for osteopetrosis with gingival hamartomas in cattle (Sartelet et al., 2014). The molecular mechanism of the observed gating alternations have however not been explained in detail and a link to the voltage-dependence or proton transport of ClC-7 has not been established. The here reported data show that the voltage gating machinery of ClC-5 regulates endosomal acidification. Moreover, it is obvious that not only the depolarization activated gating by the gating glutamate Glu_ext_ but also the gating associated with the proton glutamate Glu_in_ are important determinants of the physiological role of ClC-5. Recently, profound differences in the behavior of three mammalian CLC transporters—ClC-3, ClC-4, and ClC-5—have been demonstrated. In particular, it was shown that the different isoforms not only activate at different positive voltages but also exhibit different absolute open probabilities, i.e., different probabilities to undergo a conformational cycle associated with electrogenic ion transport and accordingly different gating charge amplitudes (compared to the corresponding ion currents). However, the physiological importance of these differences has not been demonstrated yet but has been postulated only theoretically. (Guzman et al., 2013) The experimental data reported here correlate molecular defects in ClC-5 that underlie the human renal condition Dent's disease with defects in both gating processes and establish therefore their physiological importance. It seems therefore that the gating characteristics of CLC transporters and their evolutionary optimization are an important determinant of the cellular role of these proteins.

### Conflict of interest statement

The author declares that the research was conducted in the absence of any commercial or financial relationships that could be construed as a potential conflict of interest.
